# Classification and Computational Analysis of *Arabidopsis thaliana* Sperm Cell-Specific F-Box Protein Gene *3p.AtFBP113*

**DOI:** 10.3389/fgene.2020.609668

**Published:** 2020-12-14

**Authors:** Afsheen Malik, Alvina Gul, Rabia Amir, Faiza Munir, Mustafeez Mujtaba Babar, Syeda Marriam Bakhtiar, Muhammad Qasim Hayat, Rehan Zafar Paracha, Zoya Khalid, Hadi Alipour

**Affiliations:** ^1^Department of Plant Biotechnology, Atta-ur-Rahman School of Applied Biosciences, National University of Sciences and Technology, Islamabad, Pakistan; ^2^Department of Biosciences, Shifa College of Pharmaceutical Sciences, Shifa Tameer-e-Millat University, Islamabad, Pakistan; ^3^Department of Bioinformatics and Biosciences, Capital University of Science and Technology, Islamabad, Pakistan; ^4^Research Center for Modeling and Simulation, National University of Sciences and Technology, Islamabad, Pakistan; ^5^Computational Biology Research Lab, Department of Computer Science, National University of Computer and Emerging Sciences-FAST, Islamabad, Pakistan; ^6^Department of Plant Production and Genetics, Faculty of Agriculture and Natural Resources, Urmia University, Urmia, Iran

**Keywords:** F-box protein, microgametogenesis, *Arabidopsis thaliana*, sperm cell, pollen grain, nomenclature

## Abstract

In plants, F-box proteins (FBPs) constitute one of the largest superfamilies of regulatory proteins. Most F-box proteins are shown to be an integral part of SCF complexes, which carry out the degradation of proteins and regulate diverse important biological processes. Anthers and pollen development have a huge importance in crop breeding. Despite the vast diversity of FBPs in Arabidopsis male reproductive organs, their role in anther and pollen development is not much explored. Moreover, a standard nomenclature for naming FBPs is also lacking. Here, we propose a standard nomenclature for naming the FBPs of *Arabidopsis thaliana* uniformly and carry out a systematic analysis of sperm cell-specific FBP gene, i.e., *3p.AtFBP113* due to its reported high and preferential expression, for detailed functional annotation. The results revealed that *3p.AtFBP113* is located on the small arm of chromosome and encodes 397 amino acid long soluble, stable, and hydrophilic protein with the possibility of localization in various cellular compartments. The presence of the C-terminal F-box associated domain (FBA) with immunoglobulin-like fold anticipated its role in protein binding. Gene ontology based functional annotation and tissue-specific gene co-expression analysis further strengthened its role in protein binding and ubiquitination. Moreover, various potential post/co-translational modifications were anticipated and the predicted tertiary structure also showed the presence of characteristic domains and fold. Thus, the outcomes of the study will be useful in developing a better understating of the function of *3p.AtFBP113* during the process of pollen development, which will be helpful for targeting the gene for manipulation of male fertility that has immense importance in hybrid breeding.

## Introduction

In plants, F-box proteins (FBPs) constitute one of the largest superfamilies of regulatory proteins, which control diverse biological processes from growth to development (Gagne et al., 2002; Xu et al., 2009; Abd-Hamid et al., 2020). The FBPs are more diverse in plant species as compared to other kingdoms of life and the number of genes present in their genomes reflects this diversification. For instance, in plants, Arabidopsis, Medicago, rice, maize, soybean and chickpea genomes contain 694, 972, 678, 359, 509 and 285 FBP genes, respectively (Xiao and Jang, 2000; Jain et al., 2007; Jia et al., 2013, 2017; Gupta et al., 2015; Song et al., 2015). In comparison, human, fruit fly, and *Caenorhabditis elegans* genomes contain only 38, 23 and 326 FBP genes, respectively, where *C. elegans* is the only one among all animal species whose genome contains the largest number of FBP genes (Kipreos and Pagano, 2000). Despite the presence of numerous FBP genes in the plants, a precise and standard nomenclature for naming them is lacking and the current classification system is either adopted from non-plant organisms based on C-terminal domain diversification or is based on the mutant phenotype (Kuroda et al., 2002; Abd-Hamid et al., 2020). F-box proteins are usually characterized by the presence of 50-60 amino acid long, conserved F-box motif present at their N-terminal regions (Kipreos and Pagano, 2000) and, hence, named as F-box proteins after its first discovery in a human protein Cyclin F (Bai et al., 1996). On the other hand, C terminal regions of FBPs contain protein-protein interaction domains that are mostly of diverse types and classify FBPs into different subfamilies (Bai et al., 1996; Gagne et al., 2002). Some of the commonly known subfamilies in plants are leucine-rich repeats (LRRs), Kelch repeats, WD-40, TUB, actin, F-box associated (FAB), Armadillo (Am), tetratricopeptide repeats (TPRs), Jumonji (JmjC), and DEAD-like helicase (de Pozo and Estelle, 2000; Kipreos and Pagano, 2000; Gagne et al., 2002; Xu et al., 2009).

**FIGURE 1:**
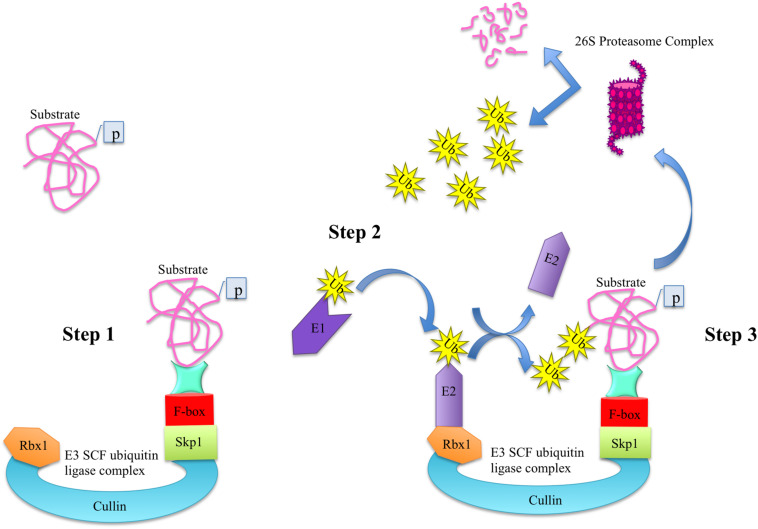
The ubiquitin-dependent protein degradation pathway. The degradation of substrate proteins via ubiquitin-dependent proteasome degradation pathway consists of three main steps. In step 1, the phosphorylated substrate is attached to the C-terminal domain of F-box protein of E3 ligase complex. In step 2, the ubiquitin-activating ligase enzyme E1 activates the ubiquitin molecules and transfers them to E2 conjugating enzyme, which is attached to Rbx1 of E3 ligase. Upon conjugation, the E3 ligase transfers the ubiquitin molecules to the substrate in step 3 and is free to enter in the second round of conjugation. Once a protein is marked with ubiquitin moieties, it is degraded into its constitute molecules via proteasome complex.

In plants, most F-box proteins are shown to be an integral part of SCF (Skp1-Cul-FBP) complexes. Among several ubiquitin-protein ligase (E3) families, SCF complex is the most dominant type of E3 ubiquitin ligases in plants that consists of four components namely S-phase kinase-associated protein 1 (Skp1), Cullin (Cul), RING-box protein 1 (Rbx1), and F-box protein (FBP). In SCF complex, the Cul acts as a core scaffolding region, the Rbx1 provides docking site to the ubiquitin-conjugation enzyme (E2) that takes activated ubiquitin molecules from ubiquitin-activating ligase enzyme (E1), Skp1 functions as a bridge and connects the FBP to Cul. The FBP imparts specificity due to its modular C-terminal protein-protein interaction domain and selectively recruits protein substrates for degradation through ubiquitin/26S proteasome pathway (UPP) in a process known as proteolysis (Figure 1; Gagne et al., 2002; Zheng et al., 2002; Cardozo and Pagano, 2004; Sadanandom et al., 2012; Sharma et al., 2016). Proteolysis or protein degradation by UPP is an important regulatory mechanism by which cells regulate their homeostasis by selectively degrading the marked proteins and hence ensure the proper functioning and adaptation of organisms in the prevailing environment (Sadanandom et al., 2012; Sharma et al., 2016). Recently, the role of UPP in anther development in Arabidopsis has been reported, where two F-box proteins namely reduced male fertility (RMF) and Secondary wall thickening-Associated F-box 1 (SAF1) as a part of SCF complex regulate anther dehiscence, pollen maturation and tapetum degeneration, respectively (Kim et al., 2010, 2012).

In seed plants, pollen grains or male gametophytes containing gametes, i.e., sperm cells develop in anthers, the male reproductive organs, during the process of male gametogenesis. The anther and pollen development and timely release of pollen grains have significant importance in crop breeding where manipulation in male fertility has been used for the generation of hybrids. However, manipulating male fertility requires a profound understanding of intricate gene networks and the underlying mechanisms essential for anther and pollen normal development (Gómze et al., 2015). Various transcriptomics studies have revealed the identification of a diverse number of genes expressed during anther and different developmental stages of pollen grains in model plant *Arabidopsis thaliana*. However, the role of only few FBP genes has been deciphered so far (Honys and Twell, 2004; Alves-Ferreira et al., 2007; Kim et al., 2008; Gusti et al., 2009; Xu et al., 2010; Pearce et al., 2015). Similarly, the analysis of sperm cell-specific transcriptome has revealed the amelioration and diversity of FBP genes in them indicating that FBPs might be playing some important role there (Borges et al., 2008). However, despite having a large number of FBPs in sperm cells their biological roles are largely obscured and unexplored. In this regard, functional annotation can provide a rapid and simple means for deciphering their potential role during anther and pollen development.

**FIGURE 2:**
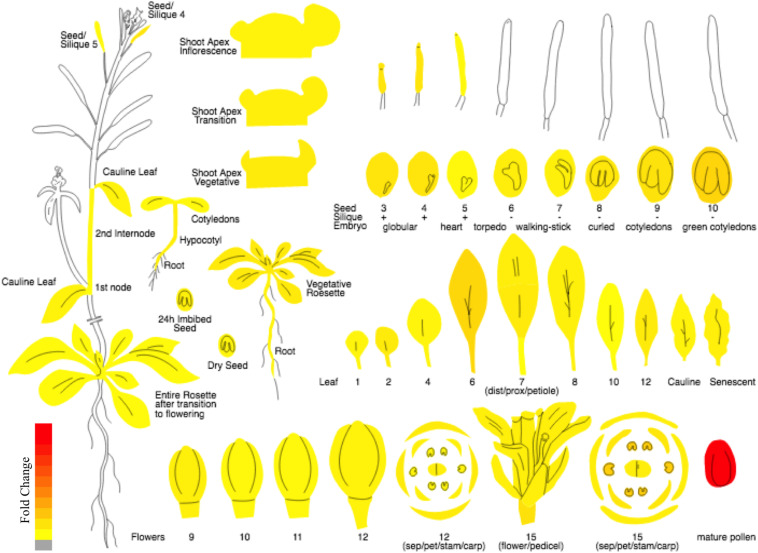
The expression atlas of *3p.AtFBP113*. The pictograph demonstrates the expression pattern of *3p.AtFBP113* across the entire plant. The *3p.AtFBP113* exhibits high and preferential expression in mature pollen. The color legend shows the expression levels in the form of fold change, where red color shows maximum and yellow color represents minimum change.

In the current study, keeping in view the need for a proper nomenclature for plant FBP genes, we propose a standard nomenclature for naming the FBP family members of *Arabidopsis thaliana*. Moreover, due to exhibiting high and preferential expression in pollen sperm cells as indicated by microarray and RT-PCR data (Borges et al., 2008) and as shown by Plant eFP viewer^1^ (Figure 2), we carry out systematic analysis and characterization of *3p.AtFBP113* in detail. Using various computational approaches, cellular localization, physicochemical properties, domains and fold architectures, protein interaction and gene co-expression networks, post-translational modifications, secondary and tertiary structures of sperm cell-specific FBP gene *3p.AtFBP113* were determined for developing a better understating of its probable function and role during male gametogenesis and pollen development.

## Materials and Methods

### Nomenclature of F-Box Protein Gene Family and Genomic Distribution

The huge diversity of FBPs in plants and their classification based on the presence of diverse C-terminal domains into different subfamilies or naming after their mutant phenotypes (Abd-Hamid et al., 2020) urge for an accurate and standardized system for naming the FBP genes. For this purpose, we propose a standard nomenclature for naming the Arabidopsis FBP genes uniformly irrespective of their C-terminal domains and mutant phenotypes. It consists of chromosome number and the arm where the gene is residing followed by the name of the plant, FBP as root name, and the serial number of the gene present on the chromosome in question according to the GenBank database^2^ (Malik, 2011). However, in case, where the same locus produces different transcripts as a result of alternative splicing the lower-case letters after gene name will distinguish among them and as a general rule, the proteins will take the name of their corresponding genes. The chromosomal localization of FBP genes was determined with the help of ePlant chromosome viewer available at BAR tools^3^ while the orientation of *3p.AtFBP113* was determined using the GenBank database available at (see text footnote 2), respectively, by using gene identifier At3g49450. The eplant tool provides a user-friendly uniform interface for visualization of data by connecting to various web services available publically (Waese et al., 2017). The genomic distribution of FBP genes was carried out using Ensembl Plants^4^.

### Subcellular Localization

The protein sequence of *3p.AtFBP113* in FASTA format was retrieved from the UniProt database^5^ using accession number Q9SCL2 for detailed analysis using computational tools. Eukaryotic cells have distinct subcellular compartments, and proteins function optimal in its subcellular localization. Therefore, the correct sorting of proteins to their final destination is imperative for performing functions (Dönnes and Höglund, 2016). Different prediction tools like DeepLoc-1.0^6^, CELLO^7^, SUBA^8^, SingalP^9^, TargetP 2.0^10^ and cNLS Mapper^11^ were used for determining the subcellular localization of 3p.AtFBP113. DeepLoc 1.0 server is an algorithm, which uses sequence information of proteins and predicts their subcellular localization using deep neural networks (Armenteros et al., 2017). CELLO uses a support vector machine (SVM) system operating at two levels (Yu et al., 2006). SUBA is a comprehensive database of published and extensive subcellular proteomics datasets, which are manually curated (Hooper et al., 2017). SignalP 5.0 server works by employing deep neural network method for predicting signal peptides and the potential residues in the protein sequences where cleavage occurs (Armenteros et al., 2019a). TargetP 2.0 server predicts transit peptides like N-terminal signal peptide, thylakoid and mitochondrial transit peptides using deep neural networks based approaches (Armenteros et al., 2019b). The cNLS Mapper predicts the nuclear localization signals in protein sequence by combining the amino acids activity profile with an additive motif scoring algorithm (Kosugi et al., 2009). Moreover, the susceptibility of 3p.AtFBP113 for being transmembrane (TM) protein was determined using online available tools like TMHMM server v. 2.0^12^ HMMTOP^13^ and Protter^14^. TMHMM is an online tool, which predicts the presence of TM helices using a hidden Markov model (HMM) (Krogh et al., 2001). HMMTOP predicts transmembrane helices and protein topology based on the differences in amino acid composition across the protein sequence using the hidden Markov model (Tusnády and Simon, 2001). The visual presentation of 3p.AtFBP113 protein topology was obtained using Protter (see text footnote 14). Protter is a web-based application for protein topology visualization by the integration of annotated and predicted protein sequences with experimental data (Omasits et al., 2014).

### Computation of Physicochemical Properties

Several physicochemical properties of 3p.AtFBP113 protein like molecular weight, isoelectric point (pI), the total number of residues bearing positive charges and negative charges, instability index, aliphatic index, and grand average of hydropathicity (GRAVY) were theoretically determined with the ProtParam tool available at ExPASy^15^.

### Functional Domain and Family Prediction

In order to predict conserved domain, motifs and family of 3p.AtFBP113 protein, a number of resources were utilized. They included Conserved Domain Database (CDD)^16^, InterProScan^17^, Conserved Domain Architecture Retrieval Tool (CDART)^18^, Simple Modular Architecture Research Tool (SMART)^19^, ScanProsite^20^, Pfam^21^, and SUPERFAMILY^22^. CDD is a resource of physically curated protein domain models, which uses 3-dimensional structure information for describing sequence-structure-function relationships (Marchler-Bauer et al., 2011). InterProScan tool allows the scanning of sequences against the InterPro signatures collected from different databases (Jones et al., 2014). CDART searches protein similarities against NCBI Entrez Protein Database using profiles of protein domains and scores them based on the domain architecture (Geer et al., 2002). SMART is a resource of manually curated protein domain models, which identifies, annotates and explores the architecture of protein domains (Letunic et al., 2015). ScanProsite is a database that contains PROSITE patterns and profiles for detection of signature motifs and protein domains on the basis of which it classifies given protein sequences into families and identifies its function (de Castro et al., 2006). Pfam database contains a great collection of protein families, their annotation and multiple sequence alignment based on hidden Markov models (HMM) (El-Gebali et al., 2019). SUPERFAMILY is a database of the Hidden Markov Model (HMM) library, which annotates proteins structurally and functionally and it classifies protein structure domains at superfamily level using Structural Classification of Protein (SCOP) database (Oates et al., 2015).

### Detection of Motifs and Folds

Motifs are short patterns of sequences that usually impart significant structural or functional characteristic to proteins and are usually conserved in protein families or subfamilies (Mohamed et al., 2016). MotifFinder^23^ was used to detect the motifs present in 3p.AtFBP113 sequence. Folds are the general architecture of proteins and are detected using the PFP-FunDSeqE predictor web server^24^. The PFP-FunDSeqE web server predicts the folds in protein sequences by coupling information of the functional domain and sequential evolution (Shen and Chou, 2009).

### Post/Co-translational Modifications Prediction

The N-, O- and C-linked glycosylation in 3p.AtFBP113 protein sequence was predicted using GlycoEP^25^ tool with 0 threshold values. This tool uses SVM models, which were developed using non-redundant datasets of glycosite patterns for the prediction of N-, O-, and C-linked glycosites with high accuracy (Chauhan et al., 2013). NetAcet 1.0 server^26^ and NetPhos 3.1 server^27^ were used for prediction of potential N-acetylation and phosphorylation sites, respectively. NetAcet server makes predictions using neural network method (Kiemer et al., 2005), while the NetPhos server predicts phosphorylation at serine, threonine and tyrosine using an artificial neural network method (Blom et al., 1999).

### Gene Ontology Based Functional Annotation

Gene ontology (GO) is a well-known tool for annotating and describing the functional attributes of genes, the biological processes in which they are involved and the cellular components they are residing in Butte and Chen (2013). The functional characterization of *3p.AtFBP113* was carried out using the ARGOT 2.5 web server^28^. ARGOT2.5 web server infers the sequence function by integrating the GO terms clustering with the weighting scheme (Fontana et al., 2009).

### Protein–Protein Interaction Network

In the cellular environment, a protein usually interacts with many other proteins for the execution of its function, therefore, the prediction of protein functional network is important to gain insight into its function (Szklarczyk et al., 2019). STRING database^29^ with a score cut-off value of 0.400 was used for predicting interacting partners of 3p.AtFBP113 protein to infer about its probable functions.

### Analysis of Gene Co-expression Profile

The Expression Angler BAR tool (see text footnote 3) was used for analyzing, exploring, interpreting, and visualizing the microarray or RNA-seq data to comprehend the probable function of *3p.AtFBP113*. The Expression Angler BAR tool identifies genes, which co-express or exhibit comparable expression patterns by calculating the correlation coefficient for expression patterns of all expression vectors of genes to the expression pattern of gene of interest (Austin et al., 2016). In Expression Angler, the microgametogenesis developmental phase was selected and searched for genes exhibiting expression patterns similar to *3p.AtFBP113* during the process. Their expression levels were obtained using the default parameter.

### Secondary Structure Prediction

The PSIPRED web tool^30^ was used for the prediction of the secondary structure of 3p.AtFBP113. PSIPRED is Position Specific Iterated-Blast based secondary structure prediction tool. It uses two feed-forward neural networks on PSI-Blast alignment output for prediction of secondary structures like alpha helixes, Beta pleated sheets and coils from the primary sequence of the protein (McGuffin et al., 2000).

### Tertiary Structure Prediction and Validation

**FIGURE 3:**
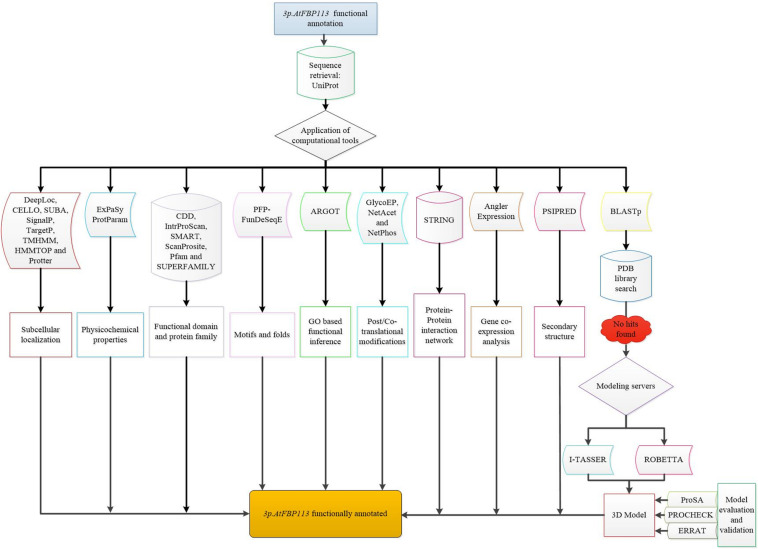
Flowchart demonstrating the functional annotation *of 3p.AtFBP113*. The diagrammatical representation depicts the comprehensive step by step methods used for inferring the probable functions of *3p.AtFBP113.*

For determining the 3-dimensional (3D) structure of 3p.AtFBP113, first protein BLAST was used for finding suitable homologous protein structure (with similarity > 30%) as a template in protein data bank library (PDB)^31^. Since the search did not yield any suitable template, so I-TASSER^32^ and Robetta^33^ servers were used for the prediction of 3p.AtFBP113 structure and function. I-TASSER server implements TASSER algorithms iteratively for the automated prediction of structure and function of target proteins using their amino acid sequences (Zhang, 2008). Robetta server analyzes the sequence and divides them into presumed domains and if the template is found comparative modeling is initiated otherwise the structure is build *ab initio* (Kim et al., 2004). Both servers return models with confidence scores. For I-TASSER generated model the C-score is from -5, 2 and for Robetta it ranges from 0 to 1, where the more positive value indicates a high reliability of models. The selected models were refined with the help of YASARA (Yet Another Scientific Artificial Reality Application) energy minimization server^34^. YASARA minimizes the energy by employing the YASARA force field (Krieger et al., 2009) and returns the output with energy and Z scores. The lower energy and higher Z score validate the structures. The 3D models were validated using ProSA, a web-based tool, which produces a Z score. The negative Z score describes that the overall quality of the model is good (Wiederstein and Sippl, 2007). The stereochemical qualities of the models were analyzed using the PDBsum module PROCHECK (Laskowski et al., 1993) and ERRAT module of SAVES v5.0 tool (Colovos and Yeates, 1993). Finally, the models were visualized using open source software PyMol (DeLano, 2002). The comprehensive flowchart describing the functional annotation of *3p.AtFBP113* is shown in Figure 3.

## Results

### Nomenclature and Genomic Distribution of *Arabidopsis thaliana* F-Box Protein Gene Family

**FIGURE 4:**
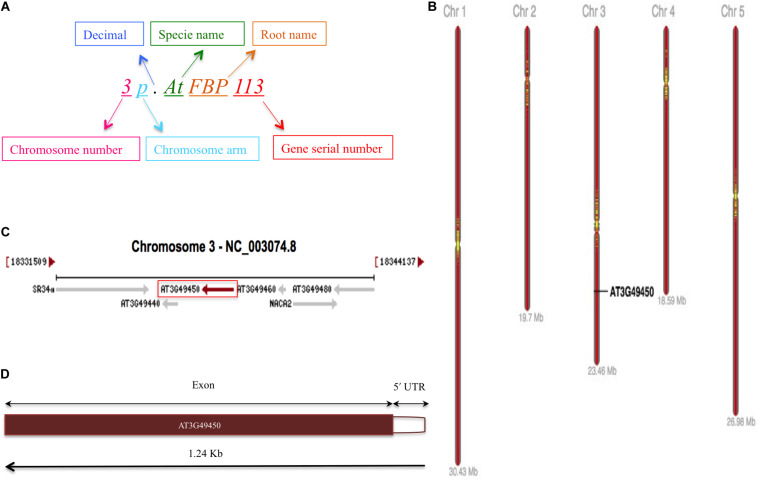
FBP gene family nomenclature system, and *3p.AtFBP113* chromosome localization, orientation and, gene structure. **(A)** Demonstrates an alphanumerical nomenclature system consisting of several parts, exemplifying gene of interest, for naming FBP gene family in Arabidopsis. **(B)** Specifically demonstrates that *3p.AtFBP113* is located on the short arm of Arabidopsis chromosome 3 in high gene density region. **(C)** Shows that *3p.AtFBP113* is located in reverse orientation on NCBI reference chromosome sequence NC_003074.8 along with other genes. **(D)** Describes *3p.AtFBP113* structure. The gene is 1.24 Kb long and encodes one exon with the 5′ UTR region.

**FIGURE 5:**
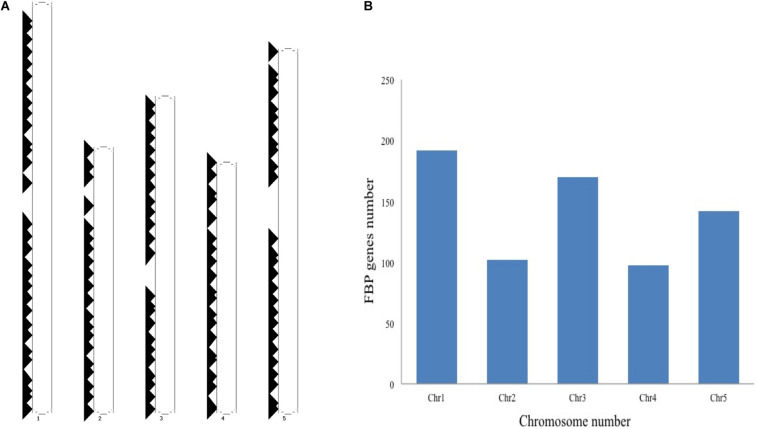
Genomic distribution of *Arabidopsis thaliana* F-box genes. **(A)** Shows the distribution and localization of FBP genes on five different chromosomes of *Arabidopsis thaliana*. **(B)** Describes the positive correlation between the chromosome length and the number of FBP encoding genes. The longer chromosomes tend to have more FBP genes as compared to the smaller chromosomes except that more FBP genes are residing on chromosome 3 as compared to chromosome 5, which is larger than chromosome 3.

The schematic representation of standardized nomenclature, which is proposed here for naming the FBP genes, exemplifying gene of interest is shown in Figure 4A, while Arabidopsis FBP gene family nomenclature is provided in Supplementary Table S1. The ePlant chromosome viewer used for determining the location of the genes revealed that our target gene is residing on the short arm (p) of chromosome 3 (Figure 4B) and according to the nomenclature our gene of interest is as *3p.AtFBP113*. The NCBI search showed that *3p.AtFBP113* is located in reverse orientation on chromosome 3 reference genome sequence NC_003074.8 (Figure 4C) while sequence analysis showed that *3p.AtFBP113* is 1.24 Kb long gene that consists of a single exon (Figure 4D) and encodes 397 amino acid long protein. The genomic distribution of FBP genes showed their even distribution on all five chromosomes (Figure 5A). The even distribution of FBP encoding genes describes a positive correlation with chromosomes length, i.e., the chromosomes 1, 3 and 5, which are longer harbor more FBP genes as compared to chromosomes 2 and 4, which are shorter in size. However, among longer chromosomes, the number of FBP encoding genes on chromosome 3 is greater as compared to chromosome 5, which is comparatively bigger in size (Figure 5B).

### Predicted Subcellular Localization and Physicochemical Properties

**FIGURE 6:**
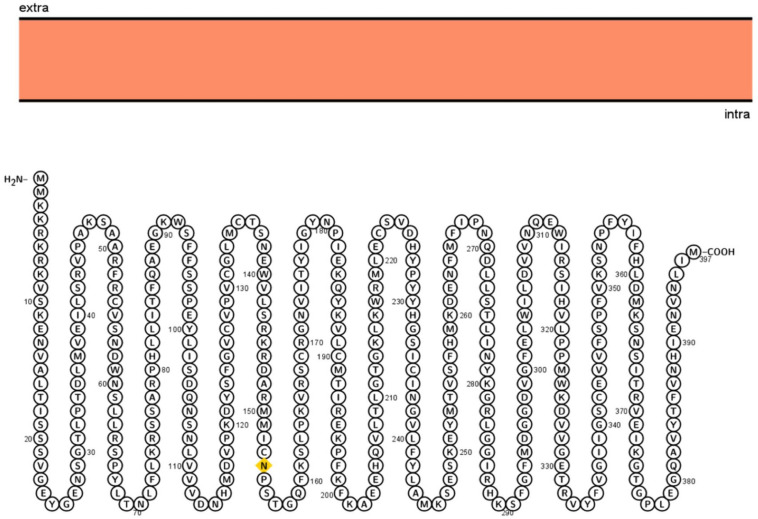
Visualization of 3p.AtFBP113 protein topology. Protter shows that 3p.AtFBP113 is lacking transmembrane helices and is present in the intracellular region. Therefore, it is deprived of the typical topology, which is the characteristic feature of TM proteins.

Different computational tools were used to perform detailed systemic analysis on the 3p.AtFBP113 protein sequence retrieved from UniPort. The subcellular localization prediction tools anticipated that 3p.AtFBP113 is located either in the nucleus, cytoplasm, or plasma membrane and lacks signal/transit peptides and transmembrane helixes. The cNLS mapper predicted the presence of two kinds of nuclear localizing signals, i.e., monopartite and bipartite. The monopartite signal, present at the N-terminal region, suggested strong nuclear localization due to high score while the bipartite signal score suggested its localization in both compartments, i.e., cytoplasm and nucleus. The visualization of 3p.AtFBP113 protein topology obtained using Protter is presented in Figure 6. The computed physicochemical properties of 3p.AtFBP113 protein are summarized in Table 1.

**TABLE 1:** Physicochemical properties of 3p.AtFBP113 protein predicted using the ProtParam tool available at the ExPASy server.

Parameters	3p.AtFBP113
Number of amino acids/protein length	397
Molecular weight (kDa)	45.432
Theoretical Isoelectric point (pI)	9.07
Total number of residues that are negatively charged (Asp + Glu)	37
Total number of residues that are positively charged (Arg + Lys)	48
Extinction coefficient (at 280 nm in H_2_O) assuming all pairs of Cysteine residues form disulfide bridges	62965 M^–1^cm^–1^
Extinction coefficient (at 280nm in H_2_O) assuming all pairs of Cysteine residues in their reduced form	62340 M^–1^cm^–1^
Instability index	39.22
Aliphatic index	84.81
Grand average of hydropathicity (GRAVY)	−0.129

### Predicted Functional Domains, Motifs, Folds, and Family

**FIGURE 7:**
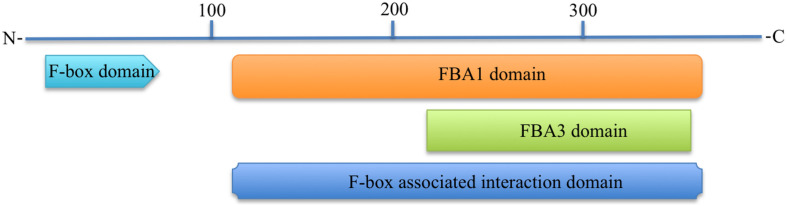
Predicted functional domains in 3p.AtFBP113 protein sequence. The figure depicts that 3p.AtFBP113 sequence contains two main domains one residing in its N-terminal region, whereas the other is present at the C-terminal region. Though different C-terminal domains are predicted from different domain prediction databases but these have overlapping regions and belong to the same subclass of F-box proteins, i.e., F-box associated domain (FBA).

All functional domain prediction databases used like CDD, ScanProsite, SMART, Pfam, CDART, and InterProScan predicted that the 3p.AtFBP113 contains an approximately 40-50 amino acid long F-box domain at its N-terminal region. Besides the N-terminal domain, some of these databases like CDD, SMART, Pfam and CDART outputs showed an additional domain at the C-terminal region. For instance, CDD, and CDART predicted F-box associated domain type1 (FBA1) spanning from 127 to 365 amino acids, while SMART and Pfam outputs showed a F-box associated domain type3 (FBA3) spanning from 233 to 357 amino at C-terminal. InterProScan database search revealed that 3p.AtFBP113 sequence contains at its C-terminus region either F-box associated interaction domain (127-365 aa) or FBA3 domain (234-357 aa). The CDD and CDART predicted it to be a member of either F-box-like domain superfamily or FBA1 domain superfamily. The InterProScan output showed it as a member of either F-box domain superfamily or F-box-like domain superfamily, whereas the Superfamily outcome anticipated it to belong to F-box domain superfamily of proteins. The domains, which are predicted by different bioinformatics tools, are shown in Figure 7. The MotifFinder detected three motifs in the protein sequence, i.e., F-box or F-box-like motif in the N-terminal region while FBA3 motif in the C-terminal region, whereas, PFP-FunDSeqE web server predicted immunoglobulin-like fold in the protein sequence.

### Predicted Post/Co-translational Modifications

**FIGURE 8:**
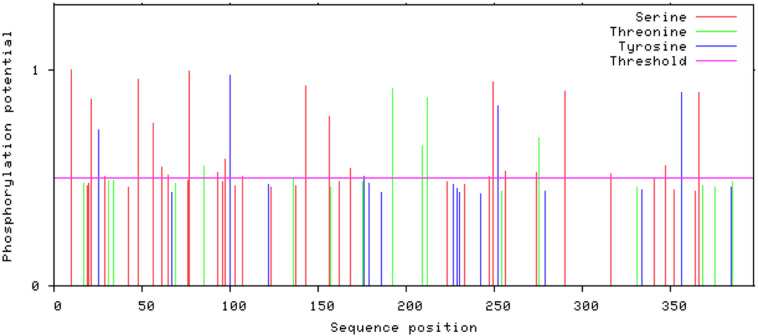
The predicted potential phosphorylation sites in 3p.AtFBP113 sequence. Several residues with high potential for phosphorylation were predicted by the NetPhos server as indicated by their high score in the graph. The higher score, i.e., 1 demonstrates high confidence in the prediction. Among these, serine residues seem to have more potential for phosphorylation as many serine residues have a score near 1.

Glycosylation is one of the major modifications of proteins after their translation and glycoproteins carry out various important processes at the cellular level like folding of proteins, cell to cell interaction, cell recognition, and host-pathogen interaction (Chauhan et al., 2013). In 3p.AtFBP113 protein sequence, the GlycoEP tool predicted 154N as a potential residue for N-linked glycosylation; many residues, i.e., 42S, 61S, and 252S were predicted as potential O-linked sites while it could not predict any potential C-linked glycosylation sites in the protein sequence. N-terminal acetylation is the most common modification, which occurs co-translationally (Kiemer et al., 2005). NetAcet 1.0 server output showed that 3p.AtFBP113 sequence does not contain any potential acetylation site. Phosphorylation at serine, threonine, or tyrosine is the most important modification, which affects signaling of various cellular processes (Blom et al., 1999). NetPhos 3.1 server predicted various potential phosphorylation sites in 3p.AtFBP113 sequence (Figure 8). The anticipated potential sites in 3p.AtFBP113 sequence for different post/co-translational modifications are mentioned in Table 2.

**TABLE 2:** Potential residues predicted in 3p.AtFBP113 protein sequence to undergo post/co-translational modifications.

Prediction Webservers	Post/Co-Translational Modifications	Potential Residues
GlycoEP	N-Linked Glycosylation	154N
	O-Linked Glycosylation	42S, 61S, 252S
	C-Linked Glycosylation	None
NetPhos 3.0	Phosphorylation	10S, 21S, 29S, 48S, 56S, 61S, 65S, 77S, 93S, 97S, 107S, 143S, 156S, 168S, 247S, 249S, 256S, 274S, 290S, 316S, 347S, 366S, 85T, 136T, 192T, 209T, 212T, 275T, 25Y, 100Y, 176Y, 252Y, 356Y
NetAcet 1.0	Acetylation	None

### Inferred Molecular and Cellular Functions Based on GO Terms, PPI Network and Gene Co-expression Analysis

ARGOT2.5 output returned various GO terms (Supplementary Table S2) among which the most significant terms anticipated that *3p.AtFBP113* is involved in the biological processes like protein ubiquitination. At the molecular level, *3p.AtFBP113* is predicted to take part in protein binding, while at the cellular level it is anticipated to be a part of the proteasome core complex.

**FIGURE 9:**
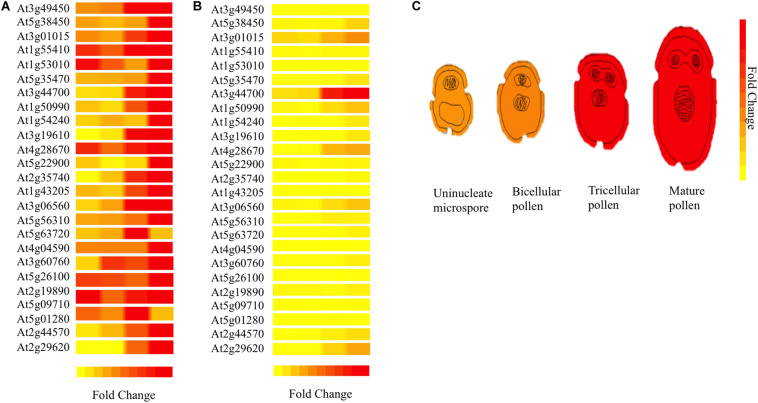
Heat map of genes co-expressed with *3p.AtFBP113* in a tissue-specific manner. The part **(A)** of the figure shows the local expression patterns of genes co-express with candidate gene *3p.AtFBP113* (At3g49450) in the form of a heat map during different developmental stages of microgametogenesis. **(B)** Shows the global view of gene expression indicating strong differential expression of a gene during last stage of microgametogenesis. The color legend indicates the expression levels in the form of fold change, where red shows maximum and yellow represents minimum change. **(C)** Depicts the expression of *3p.AtFBP113* exclusively during different developmental stages of microgametogenesis. The gene expression is low during early phases while reaches to a high level during later stages of reproduction as indicated by the color legend of fold change.

For understanding *3p.AtFBP113* functions in the context of a well-organized biological network in the cellular environment, the STRING database was used for predicting the interacting partners of 3p.AtFBP113 protein. However, with cut-off value 0.04, we could not obtain reliable interacting protein partners. Next, using Expression Angler we obtained about 24 genes having similar expression patterns with that of *3p.AtFBP113* during the process of microgametogenesis (Figure 9). TAIR database was searched for annotation of genes functions. The genes that showed strong similar expression patterns, i.e., having Pearson correlation coefficient ≥ 0.7 were mainly those involved in protein phosphorylation, cell division, transportation, protein ubiquitination, and protein binding (Supplementary Table S3).

### Secondary and Tertiary Structures of 3p.AtFBP113

**FIGURE 10:**
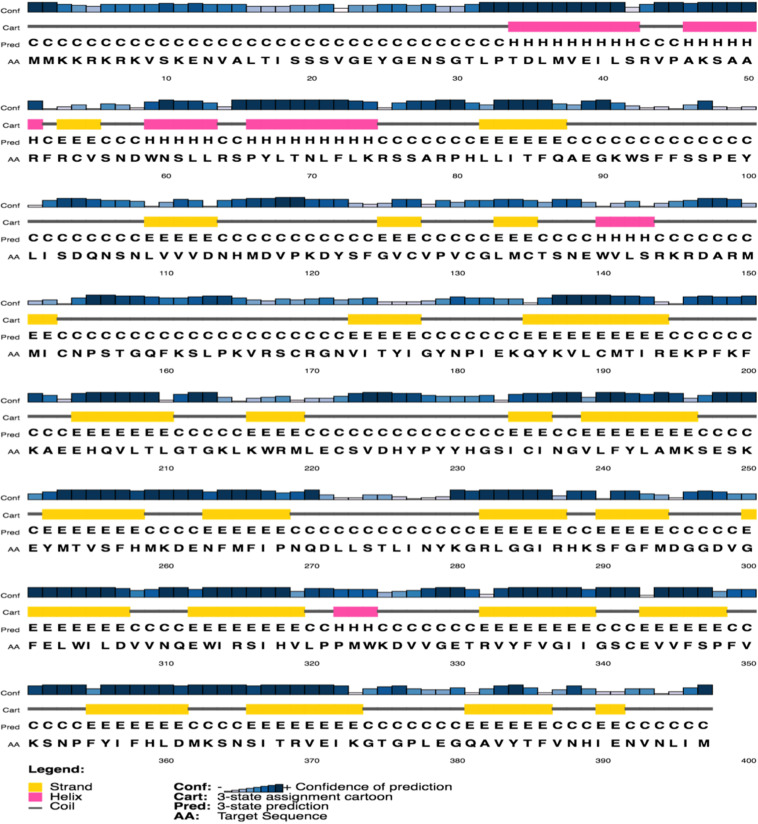
Predicted secondary structure of 3p.AtFBP113 protein. The secondary structure of 3p.AtFBP113 shows that the β-strands (yellow cylinders) are the most abundant secondary structural elements followed by α-helices (pink cylinders). Moreover, the coils (gray lines) are also abundant in the secondary structure of 3p.AtFBP113.

**FIGURE 11:**
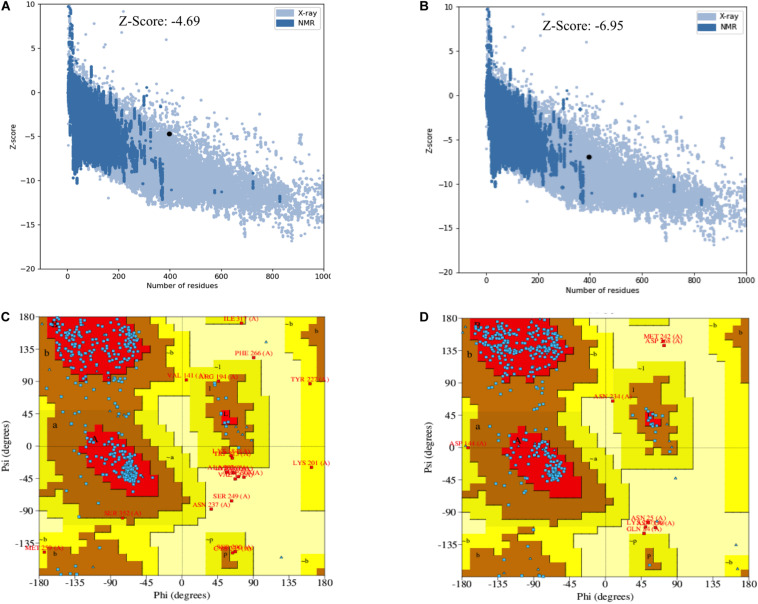
Validation of I-TASSER and Robetta generated 3p.AtFBP113 protein 3D models. The plot in part **(A)** shows Z-score for I-TASSER generated 3D model, whereas in **(B)** for the model developed by Robetta server. The graphs demonstrate good overall quality of the models due to the presence of their Z scores within the range of experimentally determined protein structures. The part **(C)** of the figure shows the phi (Φ) and psi (Ψ) bonds conformation of the 3D structure generated by I-TASSER where total 97.4 % of residues phi (Φ) and psi (Ψ) bonds are in conformation, which is either most favorable or is allowed, while 2.6% residues are outliers. **(D)** Shows the Ramachandran plot of Robetta generated 3D model. The plot shows that most favored, additionally allowed and generously allowed regions have 98% of residues while 2% of the residues have allosteric hindrances and are outliers.

**FIGURE 12:**
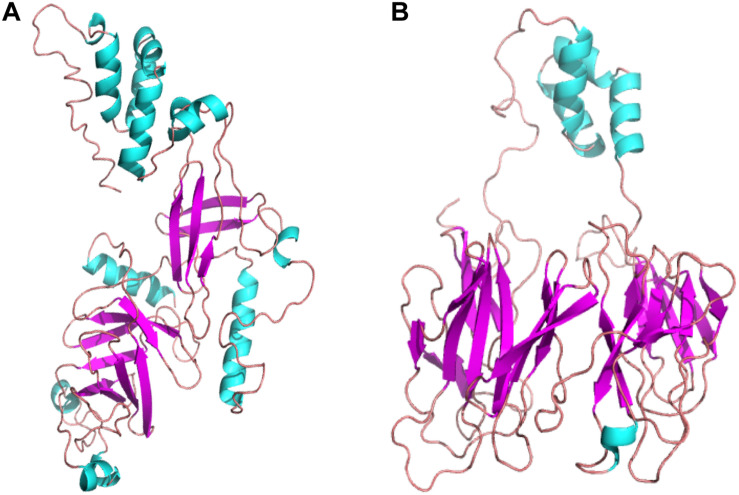
The tertiary structures of 3p.AtFBP113 protein generated by two different modeling servers. **(A)** The schematic representation of the complete tertiary structure generated by I-TASSER as a ribbon diagram. **(B)** The ribbon diagram of the 3D structure, generated by Robetta software. In both tertiary structures, the β-pleated sheaths are shown by magenta color and cyan color describes the α-helices, whereas coils (light red) constitute the rest of the protein part. The α-helices form the F-box domain in the N-terminal region, whereas two antiparallel β-pleated sheaths, which form immunoglobulin-like fold, constitute the C-terminal FBA domain.

The secondary and tertiary structures of 3p.AtFBP113 were predicted to get insights into its structural features and function. PSIPRED results showed that 3p.AtFBP113 sequence is dominated by β-pleated sheets and coils followed by α-helices (Figure 10). Since we could not find any suitable homologous protein 3D structure in the PDB library for homology modeling, so we generated 3D structures using I-TASSER and Robetta servers. I-TASSER output generated 5 models with different C-scores ranging from -4 to -2, and the model with the highest C-score that reflects high confidence was selected as a final model. The Robetta server used comparative modeling and also generated 5 models among which the best model is selected. The refinement with the YASARA energy minimization server returned the models with low energy and high Z-values. Next, the validation of I-TASSER and Robetta models with ProSA web server returned Z scores within the range of experimentally determined 3D structures of proteins and depicted their good overall quality (Figures 11A,B). The analysis of Ramachandran plots generated by the PDBsum module PROCHECK revealed that for I-TASSER and Robetta generated models, 94.3% and 97.7% of the residues phi (Φ) and psi (Ψ) bonds conformations were resided in most favored regions and additionally allowed regions, thus depicting the good quality of 3p.AtFBP113 3D models (Figures 11C,D). The ERRAT module of the SAVES v5.0 tool showed that the overall quality factor of the I-TASSER protein model was 87.9 while that of the Robetta model was 84.6. Thus all validation tools employed suggested the good quality of the predicted 3D model. Finally, the visualization of 3D models with PyMol showed the presence of two types of domains in 3p.AtFBP113 protein sequence (Figure 12).

## Discussion

In plants, F-box protein genes (FBPs) constitute one of the largest superfamilies of regulatory proteins. However, a standard and precise classification system for naming FBP genes is lacking in plants. In order to overcome the existing limitation, in this study, a nomenclature has been proposed for naming the FBP genes of *Arabidopsis thaliana* (Malik, 2011). The proposed systemic nomenclature will be helpful not only in introducing uniformity and communicating research findings effectively in the Arabidopsis FBP gene family but can also be applied to FBP gene families of other plant species whose genome sequencing have been completed and have well-defined chromosome maps. The genomic distribution of Arabidopsis FBPs depicts a positive relation between FBP number and chromosomal length as opposed to the study, which found out uneven FBPs distribution on cotton chromosomes (Zhang et al., 2019; Figure 5). Targeting the genes involved in anther or pollen development can manipulate male fertility, which is of immense importance in crop breeding and increasing the crop yield (Gómze et al., 2015). In plants, the prevalence of a huge number of FBP gene members and their involvement in controlling diverse growth and developmental processes make them potential targets for crop improvement. However, despite the identification of a large number of FBP genes expressed during male gametogenesis in Arabidopsis, the biological functions of only a few genes are described so far (Borges et al., 2008; Kim et al., 2008, 2010, 2012; Gusti et al., 2009) and functional roles for most FBPs are largely obscure and unknown. To bridge this gap, in the present study, a detailed analysis of the FBP gene, *3p.AtFBP113*, which specifically expressed in sperm cells, using advanced computational tools was carried out to dissect its probable functional role during the microgametogenesis and pollen development. The results demonstrated that *3p.AtFBP113* transcribes into 1.24 Kb long mRNA that consists of only a single exon and encodes 397 amino acid long protein. Proteins perform optimally in their subcellular localization and subcellular localization prediction anticipated that 3p.AtFBP113 is located either in the nucleus and/or is present in the cytoplasm or plasma membrane, which is consistence with the previous studies demonstrating diverse cellular compartments for FBP localization (Dill et al., 2004; Yan et al., 2011; Zhang et al., 2013; Zhou et al., 2015). The presence of the signal peptide in the N-terminal region of the proteins directs them to their final destination, i.e., plasma membrane or extracellular matrix (Blobel, 1980). The analysis demonstrated a lack of signal peptide in the protein sequence, thereby, eliminating its chance of being a classical secretory protein. Proteins lacking N-terminal signal peptides can have other kinds of sequences, which direct them to other cellular compartments like chloroplast, mitochondria, or peroxisomes, however, the TargetP tool could not detect such additional signal sequences. The analysis of the 3p.AtFBP113 protein sequence with cNLS mapper anticipated two different kinds of nuclear localizing signals that impact FBP localization and is in agreement with previous study results showing the importance of NLS in FBPs localization and their functions (Landry et al., 2012). The possibility of FBP to be a membrane protein was determined using various transmembrane (TM) predicting tools like TMHMM, HMMTOP, and Protter. All these prediction tools could not predict the presence of any potential TM helix in the protein sequence, which is necessary for qualifying as membrane protein thus supporting the possibility of nuclear and/or cytoplasmic localization of 3p.AtFBP113. The physicochemical properties of a protein are important not only for the execution of its functions but also provide significant information for setting up the experiment for protein isolation and crystallization (Mohanta et al., 2019). The detailed analysis of 3p.AtFBP113 protein sequence revealed that it has a molecular weight of 45 kDa and isoelectric point (pI) of 9.07 demonstrating its alkaline/basic nature (Table 1), which are important parameters for protein separation during electrophoresis (Mohanta et al., 2019). The instability index (<40), negative GRAVY score and high aliphatic index anticipated stable, hydrophilic/soluble nature, and high thermal stability of 3p.AtFBP113 (Table 1; Kyte and Doolittle, 1982; Rogers et al., 1986). Proteins are usually characterized by the presence of well-defined functional and structural units known as domains and folds respectively and a protein sequence can have one to several domains for the execution of their tasks, which classify them into diverse protein families (Schaeffer and Daggett, 2011; Punta et al., 2012). Virtually all domain prediction tools which were employed showed that the protein contains two distinct domains, i.e., F-box domain occupying the N-terminal region and FBA1/FBA3/FBA interaction domain at C-terminal region and belong to either F-box/F-box-like/FBA1 domain superfamily. Although all prediction tools successfully predicted different C-terminal domains in protein sequence, however, they occupy more or less the same positions (Figure 7). Moreover, the PFP-FunDSeqE web server output showed that the 3p.AtFBP113 sequence contains immunoglobulin-like fold, which is in agreement with previous findings (Kuroda et al., 2002). The immunoglobulin-like fold is characterized by the presence of two beta-sheets containing antiparallel beta-strands connected by disulfide bridge (Wang, 2013) and is known to involve in processes like binding and recognition of molecules (Halaby and Mornon, 1998). The prediction of the presence of immunoglobulin-like fold in 3p.AtFBA113 suggests that it may involve in protein binding and the presence of C-terminal FBA domain in 3p.AtFBA113 may function as a protein-protein interaction domain, which is a characteristic feature of many F-box proteins (Gagne et al., 2002).

Post/co-translational modifications introduce functional diversity in proteins and, therefore, regulate the cell environment (Karve and Cheema, 2011). Proteins undergo three main kinds of modifications during or after their translation namely glycosylation (the addition of sugar moiety), phosphorylation (transfer of phosphate group to the hydroxyl group of serine, threonine or tyrosine) and acetylation (transfer of acetyl group) among which glycosylation and phosphorylation are most prevalent in nature (Karve and Cheema, 2011). The results showed that 3p.AtFBP113 protein contains one potential residue for N-linked glycosylation and three potential residues for O-linked glycosylation. The proteins lacking N-terminal signal peptides do not experience glycosylation. Therefore, glycosylation of 3p.AtFBP113 in subcellular compartments, which is predicted to lack signal peptide, is not possible (West and Hart, 2017). However, the susceptibility of nucleo-cytoplasmic glycosylation cannot be neglected (West and Hart, 2017) and needs further experimental confirmation in this regard. Besides glycosylation, protein is predicted to contain 33 potential sites for phosphorylation, which include 22 Serine, 6 Threonine, and 5 Tyrosine residues (Table 2). However, residues with confidence values near 1 seem to have more potential for phosphorylation (Figure 8) and, therefore, warrant experimental analysis for determining the most suitable and significant phosphorylation site in the protein sequence.

The functional annotation of *3p.AtFBP113* via domain and motif prediction databases suggests its F-box protein with protein binding ability. The ARGOT2.5 output confirms the tentative annotation by identifying various GO terms among which the most significant terms were protein ubiquitination (GO:0016567), protein binding (GO:0005515), and proteasome complex, alpha-subunit complex (GO:0019773). In the cellular environment, the understating about biological and molecular functions a protein is performing requires the knowledge about the networks in which it is involved like PPI, gene regulation, and metabolic networks. However, reliable partners interacting with 3p.AtFBP113 could not be identified using the STRING database. Next, to further strengthen the aforementioned functional annotation in the biological context, digital co-expression analysis in a tissue-specific manner was carried to get insights into *3p.AtFBP113* probable function (Figure 9), which nowadays are increasingly used for annotating and inferring genes functions (D’Haeseleer et al., 2000; Aoki et al., 2007; Usadel et al., 2009; Morenorisueno et al., 2010; Li et al., 2015; Serin et al., 2016; Ma et al., 2018). The genes that exhibited strong similar expression patterns were mainly involved in cell division, protein phosphorylation, transportation, protein ubiquitination, and protein binding. These genes module further supports the possibility of involvement of *3p.AtFBP113* in protein binding and ubiquitination pathway during microgametogenesis. Various studies have shown that gametogenesis is a highly active process involving a large number of proteins and F-box mediated protein regulation by SCF-ubiquitin dependent protein degradation pathway during the process ensures tight regulation and progression of the cell cycle (Doronkin et al., 2003; Pagano, 2004; Genschik and Criqui, 2007; De La Chesnaye et al., 2008; Gusti et al., 2009; Kanatsu-Shinohara et al., 2014).

The knowledge of protein tertiary structure is of immense importance for understanding its molecular and biochemical functions (Chen, 2009). Due to the absence of suitable a PBD template for homology based structure prediction, I-TASSER, and Robetta servers were used for the generation of the good quality 3D structure of 3p.AtFBP113 (Figure 12). These 3D models can further be evaluated by introducing different genetic variations for determining their effects on its biological functions.

## Conclusion

In this study, a new standard nomenclature system for naming the FBP genes has been proposed, which will introduce uniformity and will be helpful in communicating the research findings effectively. Besides, the present study is the first attempt in demonstrating the functional role of the sperm cell-specific F-box protein gene using advanced computational tools, which suggests it as an important player during the process of microgametogenesis and a potential target for hybrid breeding in future works. This study will serve as a roadmap for the experimental elucidation of the aforementioned functions of *3p.AtFBP113* and its 3D structure. As to the best of our knowledge, none of the FBA domain containing F-box protein has been targeted for 3D structural resolution using X-ray crystallographic or NMR spectroscopic techniques, hence our 3D structure can serve as a starting point for unlocking the structure-function relationship for this largest subfamily of FBPs. Such efforts will not only provide better insight into *3p.AtFBP113* functions but also serve as a reference for elucidating the functions of other FBP genes expressing in sperm cells or pollen grains with the ultimate goal of improving crop yields to ensure food security.

## Data Availability Statement

The original contributions presented in the study are included in the article/Supplementary Material, further inquiries can be directed to the corresponding author/s.

## Author Contributions

AM conceived the idea, designed the project, did all the experiments, analyzed and interpreted the data, and wrote the manuscript. AG, RA, FM, MB, SB, MH, RP, ZK, and HA provided their input and critically revised the manuscript. AG supervised the study and finalized the manuscript. All authors read and approved the final manuscript.

## Conflict of Interest

The authors declare that the research was conducted in the absence of any commercial or financial relationships that could be construed as a potential conflict of interest.

## Footnotes

1 http://bar.utoronto.ca/eplant/

2 https://www.ncbi.nlm.nih.gov/

3 http://bar.utoronto.ca/

4 https://plants.ensembl.org/index.html

5 https://www.uniprot.org/

6 http://www.cbs.dtu.dk/services/DeepLoc

7 http://cello.life.nctu.edu.tw/

8 http://suba.live/

9 http://www.cbs.dtu.dk/services/SignalP/

10 http://www.cbs.dtu.dk/services/TargetP/

11 http://nls-mapper.iab.keio.ac.jp/cgi-bin/NLS_Mapper_form.cgi

12 https://services.healthtech.dtu.dk/service.php?TMHMM-2.0

13 http://www.enzim.hu/hmmtop/

14 http://wlab.ethz.ch/protter/

15 https://web.expasy.org/protparam/

16 https://www.ncbi.nlm.nih.gov/cdd/

17 https://www.ebi.ac.uk/interpro/search/sequence-search

18 https://www.ncbi.nlm.nih.gov/Structure/lexington/lexington.cgi

19 http://smart.embl-heidelberg.de/

20 https://prosite.expasy.org/scanprosite/

21 https://pfam.xfam.org/

22 http://www.supfam.org/SUPERFAMILY/

23 https://www.genome.jp/tools/motif/

24 http://www.csbio.sjtu.edu.cn/bioinf/PFP-FunDSeqE/

25 http://www.imtech.res.in/raghava/glycoep

26 http://www.cbs.dtu.dk/services/NetAcet/

27 http://www.cbs.dtu.dk/services/NetPhos/

28 http://www.medcomp.medicina.unipd.it/Argot2-5/

29 https://string-db.org/

30 http://bioinf.cs.ucl.ac.uk/psipred/

31 https://www.rcsb.org/

32 https://zhanglab.ccmb.med.umich.edu/I-TASSER/

33 http://robetta.bakerlab.org

34 http://yasara.org/servers.htm

## Supplementary Material

Supplementary Table 1 The proposed nomenclature of *Arabidopsis thaliana* F-box protein gene family.

Supplementary Table 2 Gene Ontology (GO) based functional annotation of *3p.AtFBP113* using ARGOT2.5 server.

Supplementary Table 3 Functional classification and annotation of genes co-expressed with *3p.AtFBP113* during microgametogenesis.

## Abbreviations

3D: three-dimensional
CDART: Conserved Domain Architecture Retrieval Tool
CDD: Conserved domain database
FBA: F-box associated domain
FBP: F-box protein
GO: gene ontology
SCF: Skp1-Cullin-F-box protein
SMART: Simple Modular Architecture Research Tool.
