# Supplementary material for: Classification and Computational Analysis of Arabidopsis thaliana Sperm Cell-Specific F-Box Protein Gene 3p.AtFBP113
Source: Front Genet. 2020 Dec 14;11:609668. doi: 10.3389/fgene.2020.609668 (PMC7767997; doi:10.3389/fgene.2020.609668)
Supplement: Supplementary Table 2 — Gene Ontology (GO) based functional annotation of 3p.AtFBP113 using ARGOT2.5 server. [file Data_Sheet_1.PDF]

All the Supplementary information with Tables

Table S1. The proposed nomenclature of *Arabidopsis thaliana* F-box protein gene family.
(Data given in separate MS-excel file)

Table S2. Gene Ontology (GO) based functional annotation of *3p.AtFBP113* using ARGOT2.5 server.
The ARGOT2.5 output shows GO terms in three main groups i.e. molecular function, biological process, and cellular component. The first column represents the GO terms; the second column holds GO ID describing the terms, whereas the third column represents the Total Score (TS) describing the confidence in the prediction. The TS threshold was kept ≥ 200.0 for obtaining good predictions. Only those terms with TS greater than 200 are shown here.

	GO Terms	GO ID	Total Score (TS)
**Molecular Function**	Protein binding	GO:0005515	549.400
	Ionotropic glutamate receptor activity	GO:0004970	449.882
	Endopeptidase activity	GO:0004175	385.098
	GO Terms	GO ID	Total Score (TS)
**Biological Process**	Protein ubiquitination	GO:0016567	1145.380
	Negative regulation of abscisic acid activated signaling pathway	GO:0009788	1115.840
	Stomatal movement	GO:0010118	769.346
	Response to water deprivation	GO:0009414	704.294
	Response to abscisic acid	GO:0009737	697.029
	Ubiquitination-dependent protein catabolic process	GO:0006511	526.464
	Abscisic acid activated signaling pathway	GO:0009738	246.104
	GO Terms	GO ID	Total Score (TS)
**Cellular Component**	Proteasome core complex, alpha subunit complex	GO:0019773	634.914
	Integral component of membrane	GO:0016021	514.779
	Membrane	GO:0016020	357.851

Table S3. Functional classification and annotation of genes co-expressed with *3p.AtFBP113* during microgametogenesis.
The first column represents the gene IDs, which co-express with *3p.AtFBP113*. In the second column, the r-values demonstrate the Pearson correlation coefficient, which describes the strength of co-expression. The genes with r-values higher than 0.7 are strongly co-expressed while those with r-values lower than 0.7 though do not co-express well but may provide significant insights into function. The third and fourth column represents the TAIR gene description and annotation.

Gene ID	r-value	TAIR gene description	TAIR annotation
At3g49450 (*3p.AtFBP113*)	1.000	F-box and associated interaction domains containing protein	-
At5g38450	0.800	CytochromeP450	GO Biological process: Oxidation-reduction process, trans-zeatin biosynthetic process GO molecular function: heme binding, monooxygenase activity, oxidoreductase activity, acting on paired donors with incorporation and reduction of molecular oxygen
At3g01015	0.799	Microtubule destabilizing protein60 (MDP60)	GO Biological process: Activation of protein kinase activity, cortical microtubule organization, microtubule deploymerization, regulation of mitotic spindle organization, unidimensional cell growth
At1g55410	0.781	Pseudogene of Cysteine/Histidine-rich C1 domain family protein	-
At1g53010	0.760	RING/U-box superfamily protein	GO Biological process: Protein uniquitinqtion
At5g35470	0.757	Hypothetical protein/Discontinued	-
At3g44700	0.748	Transmembrane protein	-
At1g50990	0.740	Kinase with tetratricopeptide repeat-domain	GO Biological process: Brassinosteroid mediated signaling pathway, protein

		containing protein	phosphorylation<br>GO molecular function:<br>ATP binding, protein binding, protein kinase activity
At1g54240	0.733	Winged-helix DNA-binding transcription factor family protein	GO Biological process: Chromosome condensation, negative regulation of DNA recombination, negative regulation of chromatin silencing, nucleosome assembly, nucleosome positioning, regulation of transcription, DNA-templated<br>GO molecular function:<br>double-stranded binding, nucleosomal DNA binding
At3g19610	0.723	CORTICAL MICROTUBULE DISORDERING6	GO Molecular function: microtubule binding
At4g28670	0.720	Cysteine-rich RECEPTOR-like kinase	GO Biological process: phosphorylation, protein phosphorylation<br>GO Molecular process:<br>Kinase activity
At5g22900	0.707	ARABIDOPSIS THALIANA CATION/H+ EXCHNAGER 3	GO Biological process:<br>cation transport, monovalent inorganic cation transport, regulation of pH<br>GO Molecular process: monovalent cation: proton antiporter activity, solute: proton antiporter activity
At2g35740	0.707	NOSITOL TRANSPORTER 3	GO Biological process:<br>glucose import<br>GO Molecular process: carbohydrate transmembrane transporter activity, myo-inositol: proton symporter activity
At1g43205	0.705	Transposable element gene	-
At3g06560	0.702	Poly(A) polymerase 3	GO Biological process:<br>mRNA polyadenylation<br>GO Molecular process:<br>protein binding, RNA binding, polynucleotide adenylyltransferase activity
At5g56310	0.699	Pentatricopeptide repeat (PPR)	GO Biological process:<br>RNA modification

		superfamily protein	
At5g63720	0.699	KOKOPELLI	GO Biological process: Double fertilization, pollen development
At4g04590	0.699	Transposable element gene	-
At3g60760	0.698	Hypothetical protein	-
At5g26100	0.698	Hypothetical protein	-
At2g19890	0.698	Hypothetical protein	GO Biological process: Regulation of gene expression
At5g09710	0.692	Magnesium transporter CorA-like family protein	GO Biological process: Magnesium ion transport GO Molecular process: Magnesium ion transmemebrane transporter activity
At5g01280	0.691	BASIC PROLINE-RICH PROTEIN3 encodes a microtubule-associated protein	-
At2g44570	0.690	GKYCOSYL HYDROLASE 9B12	GO Biological process: Carbohydrate metabolic process GO Molecular process: Cellulase activity, hydrolase activity, hydrolyzing O-glycosyl compounds
At2g29620	0.689	dentin sialophosphoprotei n	-
